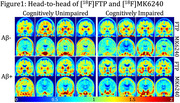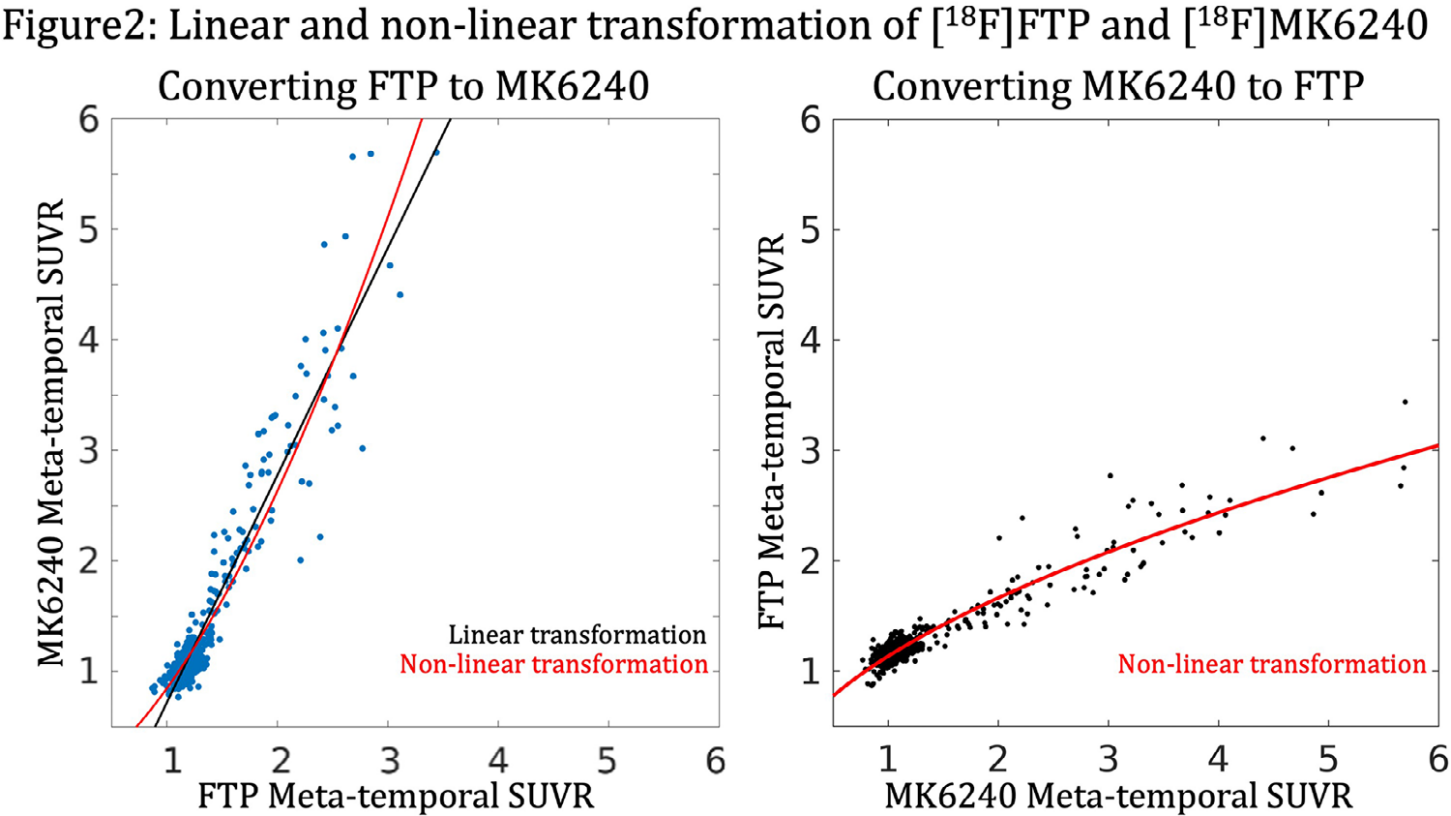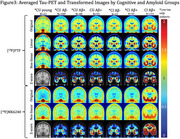# Discrepant tau‐PET signal between [^18^F]FTP and [^18^F]MK6240 in low tau individuals

**DOI:** 10.1002/alz70861_109000

**Published:** 2025-12-23

**Authors:** Cécile Tissot, Hsin‐Yeh Tsai, Renaud La Joie, Nesrine Rahmouni, Joseph Therriault, Stijn Servaes, Jenna Stevenson, Firoza Z Lussier, Arthur C. Macedo, Brian A. Gordon, Belen Pascual, Val J Lowe, David N. Soleimani‐Meigooni, Hwamee Oh, William E Klunk, Pedro Rosa‐Neto, William J. Jagust, Tharick A Pascoal, Suzanne L. Baker

**Affiliations:** ^1^ Lawrence Berkeley National Laboratory, Berkeley, CA USA; ^2^ McGill University Research Centre for Studies in Aging, Montreal, QC Canada; ^3^ Department of Neurology, University of California, San Francisco, San Francisco, CA USA; ^4^ McGill University, Montreal, QC Canada; ^5^ University of Pittsburgh, Pittsburgh, PA USA; ^6^ Washington University in St. Louis, St. Louis, MO USA; ^7^ Houston Methodist Research Institute, Houston, TX USA; ^8^ Mayo Clinic, Rochester, MN USA; ^9^ University of California, San Francisco, San Francisco, CA USA; ^10^ Brown University, Providence, RI USA; ^11^ University of California, Berkeley, Berkeley, CA USA

## Abstract

**Background:**

Tau‐PET imaging enables in vivo detection of tau pathology in Alzheimer’s disease (AD), with early detection being critical for tracking disease progression. Here, we compare [^18^F]FTP and [^18^F]MK6240 in individuals with low tau burden.

**Method:**

450 individuals from the HEAD study (22 cognitively unimpaired (CU) young (CUY), 242 CU old and 186 cognitively impaired (CI)) underwent [^18^F]FTP and [^18^F]MK6240 tau‐PET and amyloid‐PET scans. Scans were processed using identical pipelines with inferior cerebellar gray matter as the reference region. Individuals were categorized as low tau if both [^18^F]FTP and [^18^F]MK6240 meta‐temporal SUVR were lower than 1.5. For comparison, [^18^F]MK6240 and [^18^F]FTP were transformed into one another using linear and non‐linear models; additionally, images were also z‐scored relative to the CUYs.

**Result:**

Despite cognitive status and amyloid burden, several individuals showed elevated [^18^F]FTP retention in temporal regions relative to [^18^F]MK6240. As an example, Figure 1 shows [^18^F]FTP and [^18^F]MK6240 in 16 participants (4 per cognitive and amyloid status). Across most subjects with SUVRs<1.5, [^18^F]FTP presented higher retention in meta‐temporal region as compared to [^18^F]MK6240, though [^18^F]MK6240 increased sharply above this threshold (Figure 2). In Figure 3, group‐average images and their transformed counterparts are shown for each group by amyloid and cognitive status, the subset of low‐tau participants in Aβ+ groups are also shown. In low tau groups, [^18^F]FTP meta‐temporal retention is high in comparison to [^18^F]MK6240. Additionally, z‐scored [^18^F]FTP images show broader spread into temporal regions than [^18^F]MK6240, reinforcing differences in retention.

**Conclusion:**

[^18^F]FTP shows meta‐temporal retention not seen with [^18^F]MK6240 in low tau individuals. This discrepancy persists across transformed and z‐scored images, suggesting that there is a difference in early tracer retention between [^18^F]FTP and [^18^F]MK6240. It is unclear if the early [^18^F]FTP retention is related to early maturity levels of tau or off‐target signal. These findings have important implications for basic understanding of early tau retention, early diagnosis, and biomarker harmonization efforts.